# Quality of claims, references and the presentation of risk results in medical journal advertising: a comparative study in Australia, Malaysia and the United States

**DOI:** 10.1186/1471-2458-10-294

**Published:** 2010-05-29

**Authors:** Noordin Othman, Agnes I Vitry, Elizabeth E Roughead

**Affiliations:** 1 Quality Use of Medicines and Pharmacy Research Centre, School of Pharmacy and Medical Sciences, University of South Australia, Adelaide, Australia; 2 Kulliyyah of Pharmacy, International Islamic University Malaysia, Kuantan, Pahang, Malaysia

## Abstract

**Background:**

Journal advertising is used by pharmaceutical companies to disseminate medicine information to doctors. The quality of claims, references and the presentation of risk results in Australia and the US has been questioned in several studies. No recent evidence is available on the quality of claims, references and the presentation of risk results in journal advertising in Australia and the US and no Malaysian data have been published. The aim of this study was to compare the quality of claims, references and the presentation of risk results in journal advertising in these three countries.

**Methods:**

A consecutive sample of 85 unique advertisements from each country was selected from journal advertising published between January 2004 to December 2006. Claims, references and the presentation of risk results in medical journal advertising were compared between the three countries.

**Results:**

Less than one-third of the claims were unambiguous claims (Australia, 30%, Malaysia 17%, US, 23%). In Malaysia significantly less unambiguous claims were provided than in Australia and the US (P < 0.001). However, the unambiguous claims were supported by more references than other claims (80%). Most evidence was obtained from at least one randomized controlled trial, a systematic review or meta-analysis (Australia, 84%, Malaysia, 81%, US, 76%) with journal articles being the most commonly cited references in all countries. Data on file were significantly more likely to be cited in the US (17%) than in Australia (2%) and Malaysia (4%) (P < 0.001). Advertisements that provided quantitative information reported risk results exclusively as a relative risk reduction

**Conclusions:**

The majority of claims were vague suggesting poor quality of claims in journal advertising in these three countries. Evidence from a randomized controlled trial, systematic review or meta- analysis was commonly cited to support claims. However, the more frequent use of data that have not been published and independently reviewed in the US compared to Australia and Malaysia raises questions on the quality of references in the US. The use of relative rather than absolute benefits may overemphasize the benefit of medicines which may leave doctors susceptible to misinterpreting information.

## Background

Information on medicines is essential to help doctors ensure the safe and optimal use of medicines. Pharmaceutical advertisements in journal advertising are used by pharmaceutical companies to disseminate medicine information to doctors [1]. Medicines information includes product characteristics, marketing claims and references to support claims. Evidence shows that journal advertising often provides biased information [2-4]. It also appears that doctors generally underestimate the impact of pharmaceutical promotion on their prescribing practices [5-7].

In 2004, the World Health Organization reported that 89 (46%) countries regulate pharmaceutical promotion [8]. In most countries, the control of pharmaceutical promotion is overseen by pharmaceutical companies through self-regulation [9,10]. Self-regulation is intended to complement government legislation on pharmaceutical promotion, where these are available. In some countries regulatory agencies directly regulate pharmaceutical promotion [10].

In Australia and Malaysia, promotion for prescription medicines is jointly regulated through government legislation and pharmaceutical companies' code of conducts [11,12]. The codes state that all claims must be current, accurate, balanced and not misleading. Claims should be substantiated either by approved labelling or by scientific evidence [11,12]. Despite these similarities, the regulation of pharmaceutical promotion in these two countries varies in terms of transparency, financial sanctions and monitoring of promotional materials [11,12]. Unlike Malaysia, in Australia the information on all code breaches and sanctions imposed is publicly available on the pharmaceutical companies organisation's website. The level of financial sanctions in Australia is higher than in Malaysia [11,12]. The Australian code requires proactive monitoring of a random sample of promotional materials of member companies on a regular and ongoing basis.

In contrast to the Australian code, the Malaysian code does not include any provision for monitoring promotional material [11,12]. The Malaysian code relies on pharmaceutical companies to establish and maintain appropriate procedures to ensure full compliance with relevant codes and regulations and to review and monitor all of their promotional materials [11]. A unit of the Ministry of Health Malaysia, the Malaysian Advertisements Board (MAB) is authorised by law to control advertisements on medicines with medical and/or health claims [13]. To date the MAB set out standards and scrutinises publications from the print and electronic media only for non-prescription medicines to the public [13].

In the United States (US), the governmental agency, the Food and Drug Administration (FDA) [14-16], is legally mandated to regulate pharmaceutical promotion activities. The FDA requires medicines information provided by pharmaceutical companies in journal advertising to be accurate, balanced, capable of substantiation, not misleading and adhering with applicable laws and regulations. The FDA has responsibility for overseeing materials and activities that promote prescription drugs and for identifying potential violations. The FDA may regulate violations by issuing regulatory letters. The FDA may pursue enforcement action through the Department of Justice for companies that fail to undertake specific actions in response to the regulatory letters. Enforcement action may include stopping the dissemination of materials in breach of regulations and issuing corrections of previously distributed information [14-16].

Despite the control of pharmaceutical promotion in Australia and the US, prior studies in these countries have shown that pharmaceutical companies still provide poor quality marketing claims, references and information on risk results [17-20]. In 2002, Loke et al. [17] reviewed marketing claims in 174 advertisements in six Australian medical journals. Claims were classified as unambiguous, vague, emotive and presenting non-clinical outcomes such as information on half life of medicines and biochemical markers [17]. They found that only 28% of the claims made in the advertisements presented clinical outcomes in an unambiguous way [17].

References cited to substantiate marketing claims were assessed in several studies [21]. In 1994, a review of 127 distinct advertisements in four Australian medical journals found that 15% of the advertisements provided unacceptable references including unpublished company data (data on file), as well as non-English and not easily retrievable references [18]. Nearly a decade later another Australian study noted that 14% of the advertisements were unreferenced and 64% of clinical claims were not supported by randomised clinical trials or evidence from meta-analysis [17]. In the US, a review of 438 unique advertisements from the 1999 issues of 10 American medical journals found that 29% of advertisements contained no references to support marketing claims and 19% of references were data on file [19]. Another US study, reported that only 63% of 109 advertisements provided references to support the claims [20].

The results of clinical trials of medicines can be reported in different ways: absolute risk reduction (ARR), relative risk reduction (RRR) and number needed to treat (NNT) [22]. Several studies have shown that doctors' attitudes in choosing medicines for patients varies according to the presentation of the risk results [23,24]. Doctors are more likely to recommend medicines when the presentation of benefits are presented as RRR [24]. The presentation of risk results in journal advertising has been studied in a few countries [17,25-27]. However, only one study [17] has been conducted to examine how quantitative information on benefit and harm was presented in Australian journal advertising. It found that 7% of advertisements explicitly reporting quantitative outcomes provided information as RRR and none presented it as ARR or NNT. In the US, an analysis of 43 data presentations in 33 advertisements that contained quantitative research results published in four medical journals found none of the advertisements provided risk results as NNTs [27]. The availability of information on ARR and RRR was not reported in this study [27].

To our knowledge, no study has assessed the quality of claims, references and the presentation of risk results in medical journal advertising in Malaysia. The most recent studies in Australia and the US were published in 2002 and 2005 respectively. No comparative study has been conducted on the quality of claims, references and the presentation of risk results among these three countries.

This study aimed to compare types of claims, types of references and presentation of risk results in medical journal advertising in Australia, Malaysia and the United States. These countries represent two developed and one emerging country with different regulatory frameworks and resources to control promotional activities.

The major objectives were:

1- to classify types of claims made about benefits or harms outcomes as unambiguous, vague, emotive and non-clinical claims.

2- to compare the availability of references to support claims made about benefits or harms.

3- to examine if risk results were reported as ARR, RRR or NNT.

4 - to compare results for all outcomes across countries.

The minor objectives were:

1- to determine the proportion of claims supported by references.

2- to assess the types of references cited to support claims in journal advertising

3- to examine the research design and level of evidence provided by MEDLINE^® ^references cited to support claims.

## Methods

### Selection of advertisements

We used a convenience sample of one medical journal from Australia, Malaysia and United States. Journals were selected because of their high circulation amongst general practitioners. We sampled advertisement-rich journals from Australia and the US. We also included one prescribing reference manual published in Malaysia as a preliminary survey showed that fewer advertisements were published in the only Malaysian medical journal available in three established medical school libraries in Malaysia than in Australian and US journals.

The journals selected covered primary care practitioners' publications:

-Australian Family Physician, which is the official journal of the Royal Australian College of General Practitioners (readership = 38,608 with about 28,000 of these being general practitioners) (Jonathon Tremain, personal communication 2009 Feb 02).

-American Family Physician, which is the official clinical journal of the American Academy of Family Physicians (readership = over 188,200) [28].

-MIMS, which is regarded as an official drug reference of the Malaysian Medical Association (MMA) (readership =7000, with about 4200 of these being general practitioners) (Eileen Khoo, personal communication 2009 Feb 03).

-Medical Journal of Malaysia (MJM), which is the only Malaysian medical journal that is subscribed by the three established medical schools in Malaysia, Universiti Sains Malaysia, Universiti Kebangsaan Malaysia and University of Malaya (readership = over 3500, no data are available on general practitioners' readership).

A consecutive sample of 85 unique advertisements for prescription medicines was chosen from the selected publications published between January 2004 to December 2006 from each country. A product advertisement different from other advertisements for the same product in terms of graphic presentation or written content was considered to be one unique advertisement.

We documented the claims made about benefits or harms (e.g. "Add X to a statin to achieve powerful cholesterol reduction"). A claim was defined as any marketing statement or a group of statements related to the same topic (e.g. "Durable control: Provides additive and durable glycaemia control" or "Vast clinical experience: # 1 prescribed statin in Malaysia, more than 48 million patient - years of experience, more than 400 ongoing and completed trials").

Information about the Pharmaceutical Benefits Scheme (PBS) listings appearing in Australian advertisements (e.g. "PBS Information: This product is listed on the PBS as a calcium channel blocker") was excluded because the information was authorised by the Australian government [29]. Any claim that appeared more than once in an advertisement was considered as a single claim. Medicines information provided in product information (Australia and Malaysia) or prescribing information (US) and a quote from product information was not considered as a marketing claim. Product information or prescribing information is a comprehensive document that contains information to ensure appropriate use of a medicine. Unlike marketing claims, it undergoes an extensive review process between the sponsoring companies and government regulatory bodies at the time of market approval [30,31].

Claims made about benefits or harms were classified as: unambiguous clinical outcome, vague clinical outcome, emotive or immeasurable outcome and non-clinical outcome (Table 1). This classification has been previously used in two other studies of the quality of claims in medical journal advertising [17,26].

**Table 1:** Definition of claims [17,26].

Claims	Example
A: Unambiguous clinical outcome:	When compared with DRUG X, DRUG Y delivers faster symptom relief.

B: Vague clinical outcome:	DRUG X is the new, effective pill with a low incidence of discontinuation due to skin problems.

C: Emotive or immeasurable outcome:	DRUG X - one of a kind or DRUG X - a source of healing power.

D: Non-clinical outcome (e.g. drug plasma half-lives or biochemical markers):	Using DRUG X resulted in a 30% increase in arterial luminal diameter in post-mortem dissections.

We noted whether the claims were supported by references. References cited from journal articles to support claims were retrieved and classified by study design and level of evidence according to a modification of the classification of the National Health and Medical Research Council (NHMRC) guideline of Australia (Table 2) [32].

**Table 2:** Level of evidence [44].

Level of evidence	Definition
I	Evidence obtained from a systematic review or metaanalysis

II	Evidence obtained from at least one randomized controlled trial.

III	Evidence obtained from a comparative study.

IV	Other evidence.
	-Evidence obtained from studies which did not assess clinical and public health interventions.
	- Evidence obtained from reviews.

For each claim providing quantitative information, we noted if the risk results were expressed as relative risk reduction (RRR), absolute risk reduction (ARR) or number needed to treat (NNT).

### Inter-rater reliability

All data were extracted by one researcher. Three other researchers, a researcher from Australia and a pharmacist and a family medicine specialist from Malaysia independently selected the claims made about benefits or harms and determined the availability of risk results in a randomly selected sample of 30 advertisements from each country.

Kappa tests were undertaken using STATA version 10 to assess the consistency of ratings between observers.

## Data Analysis

Data were analysed using SPSS database version 14.0. Chi-square analysis was used to assess differences between countries.

## Results

A total of 255 distinct advertisements for 136 pharmaceutical products were included.

Kappa (κ) for inter-rater reliability for the classification of unambiguous, vague, emotive and non-clinical claims was calculated as 0.74 (substantial agreement) (z = 23.7, p < 0.001) [33]. Kappa (κ) for inter-rater reliability for availability of risk results between the researchers was calculated as 0.86 (almost perfect agreement) (z = 34.4, p < 0.05) [33] (Table 3 ).

**Table 3:** Inter-rater reliability test: Distribution of claims.

Country	Number of advertisements	Unambiguous	Vague	Emotive	Non- clinical	Total
Australia	30	11	20	9	0	40

Malaysia	30	20	52	6	10	88

US	30	26	65	8	12	111

Total	90	57	137	23	22	239

### Claims

There were 829 claims about benefits or harms including 165 in Australia (median 1, range 1 -7), 346 claims in Malaysia (median 4, range 1-11) and 318 in the US (median 3, range 1-9) (Table 4).

**Table 4:** Examples of marketing claims.

Claims	Type	Reason	Brand	Company	Country
"Gardasil also helps to protect your patients against other HPV related disease and cervical lesions due to HPV types 6,11,16 and 18"	Unambiguous	Clear explanation on indication and effectiveness.	Gardasil^®^	Merck Sharp & Dohme	Australia
"Levofloxacin is US FDA approved for the once-daily treatment of respiratory tract infections, urinary tract infections....	Unambiguous	Clear explanation on dosage and indication	Cravit^®^	Daewon Pharm	Malaysia
"Well tolerated comparable to Celexa"	Unambiguous	Comparative tolerability given	Effexor^®^	Wyeth	US
"Zomig nasal spray-proven speed with significant efficacy"	Vague	Compare to which medicines?	Zomig^®^	AstraZeneca	US
"Switch to new Stilnox CR for better sleep performance"	Vague	Better sleep performance compare to what?	Stilnox CR^®^	Sanofi-Aventis	Australia
"Levitra works rapidly"	Vague	How rapid? Compare to what?	Levitra^®^	Bayer	Malaysia
"It's got the power"	Emotive	Immeasurable outcome	Nexium^®^	AstraZeneca	Australia
"When you patients need relief"	Emotive	Immeasurable outcome	Sanctural^®^	Allergan	US
"It's unique DOT matrix technology optimises drug delivery"	Non-clinical	Drug delivery information	Estradot^®^	Norvatis	Australia
"Stable in the presence of a variety of B-lactamase"	Non-clinical	Biochemical information	Spectracef^®^	Cornerstone Therapeutics	US

The majority of claims were categorised as vague claims (Australia, 46%, Malaysia, 59%, US, 49%) (Table 5). Less than one-third of the claims were categorised as unambiguous claims (Australia 30%, Malaysia 17%, US, 23%) with significantly less unambiguous claims in the Malaysian advertisements compared to Australia and the US (χ2 = 29.4; df = 6, P < 0.001).

**Table 5:** Type of claims.

Classification of claims	Australia n/165 (%)	Malaysia n/346 (%)	US n/318 (%)
Unambiguous	50	58	74
	(30)	(17)	(23)

Vague	76	205	157
	(46)	(59)	(49)

Emotive	35	52	48
	(21)	(15)	(15)

Non-clinical	4	31	39
	(2)	(9)	(12)

Australia and Malaysia		P < 0.001	

Australia and the US		P = 0.001	

Malaysia and the US		P = 0.042	

### References

We found that 19% -100% of claims were referenced (Table 6). Where reference, claims were substantiated by between one to 23 references with a median of 1 in Australia and 2 in Malaysia and the US. More references were provided in the Malaysian advertisements (n = 433) compared to the Australian (n = 244) and the US (n = 233) advertisements. Emotive and vague claims were less likely to be referenced by references than non-clinical and unambiguous claims. The most commonly cited references to support claims in all countries were journal articles [see Additional file 1]. Significantly less journal articles were cited to support claims in the US (41%) than in Australia (73%) and Malaysia (72%) (P < 0.001). Significantly more data on file were cited in the US (17%) than in Australia (2%) and Malaysia (4%) (χ2 = 174.4; df = 10, P < 0.001). All types of claims in all countries were usually supported by journal articles catalogued in MEDLINE^® ^[see Additional file 1]. In all countries most references cited were substantiated by evidence obtained from at least one randomized controlled trial. Less than 7% of claims were supported by evidence obtained from a systematic review or meta-analysis.

**Table 6:** Claims supported by references.

Claims with references	Australia **n/N**^**#**^**(%)**	Malaysia **n/N**^**#**^**(%)**	US ** n/N**^**#**^**(%)**
Unambiguous	44/50	46/58	54/74
	(88)	(80)	(73)

Vague	58/76	129/205	71/157
	(76)	(63)	(45)

Emotive	20/35	19/52	9/48
	(57)	(37)	(19)

Non-clinical	2/2	17/31	30/39
	(100)	(55)	(77)

Australia and Malaysia		P = 0.001	

Australia and the US		P < 0.001	

Malaysia and the US		P < 0.001	

# Total for each type of claim

Twenty one (21%) advertisements reported risk results. Overall, most advertisements (86%) reported risk results exclusively as RRRs.

## Discussion

This study found that the majority of claims made about benefits or harms in journal advertisements in Australia, Malaysia, and the United Sates (US) were considered vague claims. Vague claims are unlikely to help doctors to make informed decisions about the value of the medicines promoted. The paucity of evidence-based information raises concern about the educational value of advertisements.

Unambiguous claims provide clear and precise information of the promoted drugs (e.g "X cures more otitis externa patients than Y" and "Two to three times more patients maintained abstinence vs. placebo in long-and short-term studies, respectively"). These types of claims should be substantiated by scientific evidence as required by the pharmaceutical regulation in the three countries studied [11,12,14]. The failure of pharmaceutical companies to provide evidence to support some unambiguous claims in these three countries highlights the need for better control of pharmaceutical promotion with regards to the need for references to support claims.

Vague (e.g " X provides rapid sleep onset") and emotive claims (e.g "A powerful SSRI that's well tolerated") were less likely to be supported with any references. However, 45% to 76% of vague claims were substantiated by references. The use of references may make advertising more credible to readers [34,35]. Several studies have shown that information from journal advertising is one of the main sources of information for newly marketed medicines [1,36,37,39]. Clear guidelines on the use of references to support marketing claims should be developed.

In contrast with Australia and Malaysia, journal advertising in the US cited fewer journal articles and more data on file (17%). The use of data on file by pharmaceutical companies in the US is consistent with a previous finding [19] that this occurred in 19% of advertisements in 2005. While the use of data on file to substantiate pharmaceutical claims in pharmaceutical promotion is allowed by the FDA [14], data on file is unpublished and has not been independently reviewed. This raises concern about the quality of evidence supporting claims as the validity of data on file may be lower than that of published journal articles [40] and cannot be easily checked by health professionals.

We found that the majority of claims cited journal articles that were retrievable by MEDLINE^®^. MEDLINE^® ^was chosen because it is widely used by health care professionals and available freely worldwide. However, non-clinical claims in Malaysia were less likely to be substantiated by retrievable journal articles. This is not due to Malaysian journals not being indexed in MEDLINE^®^. Of the 59 irretrievable journal articles cited in Malaysian journal advertising, only one reference was published in a Malaysian journal. Given the pharmaceutical companies in Malaysia tend to cite references that were not indexed in MEDLINE^® ^or published in Malaysian journals, Malaysian health professionals might not be able to evaluate the cited references to justify the validity of claims. The Pharmaceutical Association of Malaysia (PhAMA) which administers a code of conduct as a guide for the pharmaceutical promotion in Malaysia [11] needs to strengthen its code by providing clearer guidelines on the availability of references to health professionals.

The majority of references in Australia, Malaysia and the US were supported by randomized controlled trial, systematic review or meta-analysis evidence. In Australia, there was an increase in the number of references supported by randomised controlled trials, systematic reviews or meta-analyses (84%) compared with an earlier study (45%) [17]. The use of these references was higher than reported previously in the UK (31%) [41], Finland (11%) [26] and Spain (67%) [42] in 1997, 2004, and 2003 respectively.

While only a few advertisements reported risk results, advertisements with quantitative information in Australia, Malaysia and the US presented risk results exclusively as RRR. This is consistent with the results of previous studies [17,26]. The emphasis on relative rather than absolute statistics may overstate the benefit of medicines which may lead to irrational prescribing [23,24]. The reliability of journal advertising would be improved if regulations and codes of conduct included specific requirements with regards to the presentation of quantitative information.

There are limitations to this study. The validity of our results may be limited by the small sample size and the choice of only three countries. We only surveyed advertisements published in three journals and one prescribing reference manual. Pharmaceutical companies and medical journals may have different advertising policies and target audiences. Analysis of the types of medicines by therapeutic groups advertised in these journals was found to differ between countries. Medications for cardiovascular (38/8, 41%) and respiratory diseases (15/85, 17%) were mostly advertised in Australia, cardiovascular (37/85, 44%) and infectious diseases (16/85, 19%) in Malaysia, and neurological (15/85, 17%), infectious (13/85,15%), psychiatry (12/85,14%) and cardiovascular diseases (11/85, 13%) in the US respectively. Our findings may not be generalisable to different journals or countries. However our findings are consistent with two previous studies that reviewed advertisements in four Australian primary care practitioners' publications [43] and 10 multi-disciplinary American medical journals [19]

We did not examine the accuracy of claims and did not assess whether the references adequately substantiated each claim made in the advertisements. We did not attempt to request data on file from pharmaceutical companies. This may represent a limitation as data on file might refer to a randomised controlled trial, systematic review or meta-analysis. The use of a single database to assess the retrievability of journal articles is also a potential limitation. MEDLINE^® ^may not be equally representative of the scope of information that clinicians rely on in each of the countries studied.

## Conclusion

Despite the differences in regulation of pharmaceutical promotion in Australia, Malaysia and the US, this study found that the majority of claims presented were vague. The evidence base to support claims appears to be improving with the majority of references cited providing randomized controlled trial, systematic review or meta-analysis evidence. However, the more frequent use of data that have not been published and independently reviewed in the US compared to Australia and Malaysia raises questions on the quality of references in the US. There are concerns that the use of references may make advertising more convincing to readers, even when supporting non-scientific evidence. The use of relative rather than absolute benefits may overemphasize the benefit of medicines which may leave doctors susceptible to misinterpreting information. Methods to further improve the quality of pharmaceutical promotion are still required.

## Competing interests

Two of the authors (NO and AV) and one of the reviewers (RC) are members of Healthy Skepticism, an international non-profit organisation aiming to improve health by reducing harm from misleading drug promotion.

## Authors' contributions

NO designed the study with input from AV and ER. NO reviewed the journals, identified the advertisements, gathered and interpreted the data; and drafted the manuscript. All authors were responsible for critical revision of the manuscript and approved the final version submitted.

## Pre-publication history

The pre-publication history for this paper can be accessed here:

http://www.biomedcentral.com/1471-2458/10/294/prepub

## Supplementary Material

Additional file 1 Type of references, Claims with journal articles retrievable through MEDLINE® and Level of evidence. Click here for file
