# Supplementary material for: Quality of claims, references and the presentation of risk results in medical journal advertising: a comparative study in Australia, Malaysia and the United States
Source: BMC Public Health. 2010 May 29;10:294. doi: 10.1186/1471-2458-10-294 (PMC2895591; doi:10.1186/1471-2458-10-294)
Supplement: Additional file 1 — Type of references, Claims with journal articles retrievable through MEDLINE® and Level of evidence. [file 1471-2458-10-294-S1.PDF]

Table 7: Type of references, Claims with journal articles retrievable through MEDLINE® and Level of evidence.

Outcome measures	Type of references*						Claims with journal articles retrievable through MEDLINE®				Level of evidence**			
	Aus: n/244 (%), Mal: n/433 (%), US: n/233 (%)						n/N (%)				Aus: n/159 (%), Mal: n/254 (%), US: n/90 (%)			
	JA	DOF	PI	ABS	BM	OT	Un	Vag	Emo	Non	I	II	III	IV
Australia	177 (72)	4 (2)	39 (16)	7 (3)	2 (1)	15 (6)	42/44 (96)	49/58 (85)	20/20 (100)	2/2 (100)	9 (6)	125 (78)	3 (2)	22 (14)
Malaysia	313 (72)	16 (4)	32 (7)	55 (13)	10 (2)	7 (2)	38/46 (83)	116/19 (90)	15/19 (79)	11/17 (65)	18 (7)	188 (74)	10 (4)	38 (15)
US	96 (41)	40 (17)	69 (30)	8 (3)	2 (1)	18 (8)	51/54 (94)	67/71 (94)	9/9 (100)	30/30 (100)	1 (1)	67 (75)	4 (4)	18 (20)
Aus and Mal	P < 0.001						P < 0.001				P = 0.75	P = 0.23	P = 0.24	P = 0.75
Aus and the US	P < 0.001						P < 0.001				P = 0.06	P = 0.50	P = 0.24	P = 0.20
Mal and the US	P < 0.001						P < 0.001				P = 0.03	P = 0.77	P = 0.83	P = 0.27

*Percentage calculated per total number of references, **Percentage calculated per total number of journal articles retrieved, JA : Journal article, DOF: Data on file, PI: Prescribing information, ABS: Abstract, in press, conference, BM: Book, monograph, bulletin, OT: Other, Un: Unambiguous, Vag: Vague, Emo: Emotive, Non: Non-clinical.
